# Massive intestinal infarction following retroperitoneoscopic right lumbar sympathectomy

**DOI:** 10.4103/0972-9941.28185

**Published:** 2006-12

**Authors:** Francesco Rulli, Gabriele Galatà, Chiara Micossi, Carlo Dell’Isola

**Affiliations:** Department of Surgery, University of Rome Tor Vergata, Roma, Italia

**Keywords:** Intestinal infarction, laparoscopy, retroperitoneoscopy, retro-pneumoperitoneum

## Abstract

The adverse physiological effects of pneumo and retro-peritoneum are relatively well known. However, the clinical implications of compromised mesenteric circulation through several mechanical and physiological mechanisms are not as well recognized. We describe a fatal case of intestinal infarction following an elective retroperitoneoscopic right sympathectomy. The patient was a 88-year-old man who died 30 hours after an uneventful anesthesia and right endoscopic lumbar sympathectomy. An emergency explorative laparotomy revealed a massive intestinal infarction due to thrombosis of the superior mesenteric artery. We reviewed the literature on laparoscopic procedures and mesenteric ischemia. To our knowledge, this is the first reported case of intestinal infarction following retro-pneumoperitoneum. We conclude that the presence of a severe multidistrectual? arteriopathy may represent a major risk factor in retroperitoneoscopic procedures.

## INTRODUCTION

Surgeons are aware of the adverse physiological effects of a prolonged retro- and pneumo-peritoneum for minimally invasive procedures. However, far less is known about the clinical implications of compromised mesenteric circulation, especially in patients with severe atheromatosis.

We report the case of an elderly man who died of massive intestinal infarction following an elective retroperitoneoscopic right sympathectomy. After a review of the literature, we conclude that this is the first such case to be described.

## CASE REPORT

An 88-year-old man was admitted to the hospital for an elective right retroperitoneoscopic lumbar sympathectomy for a right limb stage III (Leriche-Fontaine) arteriopathy. Two years before, the patient had been operated for a bilateral carotid endoarteriotomy and for an over knee right arterial bypass.

He used to smoke 20 cigarettes a day. His ASA grade was III. Apart from mild hypertension controlled with ACE inhibitor, he was medically fit and well. The anesthesia and surgery were uneventful. The pressure of the retro-pneumoperitoneum was 15 mmHg and the total duration of the surgery was 120 minutes. Three hours after the surgical procedure, he complained of a right abdominal pain which was unresponsive to parenteral analgesics (morphine 30 mg). The abdomen was soft on palpation and there was no distension. Though his vital signs were stable, the abdominal pain persisted. Minimal tenderness was localized in the right lower quadrant of his abdomen. His hematology and biochemistry results were all within normal limits, except for leucocytosis (WBC: 20,000/mm^3^) and LDH (880 IU/lt). His chest radiograph was normal and a supine abdominal film showed a gas-filled transverse colon without any unusual features. The worsening of the symptomatology, prompted us to undertake an exploratory laparotomy on the postoperative day 1.

Laparotomy revealed a massive intestinal infarction extending to the right and transverse colon and the small bowel; the atheromatous superior mesenteric artery lumen was completely empty. The patient died a few hours after the exploratory laparotomy.

## DISCUSSION

Experimental studies have shown that the elevation of intra-abdominal pressure by gas insufflation leads to hemodynamic alteration of the peritoneal viscera and may produce splanchnic ischemia. Although the adverse physiological effects of pneumo-peritoneum are well understood, the clinical implications of compromised mesenteric circulation through several mechanical and physiological mechanisms are not as well recognized. The effects of retro-pneumo-peritoneum are even less known. Anyway, it is well known that pneumo-peritoneum during laparoscopic surgery, produces a significant decrease in hepatic microcirculation.[1–3] A study using nasogastric tonometry demonstrated that even a pneumo-peritoneum pressure of 12-15 mmHg can cause significant splanchnic mucosal ischemia.[4,5] The severe arteriopathy, with prolonged lateral and Trendelenburg’s position, may have played a determinant role in the pathogenesis of the massive intestinal infarction in our patient.

There is a case report of “nonspecific ulcerated jejunitis” that developed a few days after laparoscopic cholecystectomy and was attributed to ischemia-reperfusion injury following an ischemic period caused by the pneumo-peritoneum,[1] but similar cases following retro-pneumoperitoneum have not been documented in the literature. It has been reported that certain preexisting conditions such as hypercoagulable states in a patient undergoing laparoscopic surgery, as well as multi-vessel splanchnic atheromatosis can increase the risk of splanchnic vessel thrombosis.[4] The risk is particularly high when the laparoscopic procedure is lengthy, as in our case. A Medline search revealed seven cases of small bowel ischemia following laparoscopic cholecystectomy.[2,3]

We believe that the presence of a multi-vessel visceral arteriopathy and the length of the retro-pneumoperitoneum led to the massive intestinal infarction. Postoperative abdominal pain, intense leucocytosis and elevated LDH as a marker of tissue breakdown, may be relevant data even in a lack of a diagnostic imaging work up (i.e., abdominal computed tomography). We strongly suggest that prolonged retro-pneumoperitoneum is probably best avoided in patients with ASA III and in those who have a diffuse arteriopathy.
